# Granulomatosis With Polyangiitis Presenting as Pulmonary Masses With Coexisting Infection: A Diagnostic Challenge Leading to Life-Threatening Pulmonary-Renal Syndrome

**DOI:** 10.7759/cureus.108470

**Published:** 2026-05-08

**Authors:** Ibrahim Saleh, Mostafa Abdelaty, Mohamed Gendia, Noha Abdelkarim, Ammar Ahmed

**Affiliations:** 1 Rheumatology, Al-Sabah Hospital, Kuwait City, KWT; 2 Respiratory Medicine, Al-Sabah Hospital, Kuwait City, KWT; 3 Nephrology, Al-Sabah Hospital, Kuwait City, KWT

**Keywords:** anca-associated vasculitis, diffuse alveolar hemorrhage, granulomatosis with polyangiitis, lung mass, pulmonary-renal syndrome

## Abstract

Granulomatosis with polyangiitis (GPA) is an antineutrophil cytoplasmic antibody (ANCA)-associated vasculitis that can mimic infection and malignancy, posing a significant diagnostic challenge. We report the case of a 39-year-old male smoker with a strong family history of lung cancer who presented with fever, weight loss, dyspnea, and hemoptysis. Initial evaluation supported infection, with sputum cultures positive for *Pseudomonas aeruginosa* and *Haemophilus influenzae*. Imaging later revealed pulmonary masses suspicious for malignancy. The patient subsequently deteriorated with massive hemoptysis and acute kidney injury, requiring ICU admission, mechanical ventilation, blood transfusion, and dialysis. Further investigations demonstrated proteinase 3-ANCA positivity and diffuse alveolar hemorrhage on bronchoalveolar lavage, confirming GPA. Treatment with pulse corticosteroids, IV cyclophosphamide, and plasma exchange resulted in marked clinical recovery. This case underscores the significant diagnostic challenge posed by the overlap between infection, malignancy, and ANCA-associated vasculitis, highlighting the importance of early recognition and timely initiation of immunosuppressive therapy.

## Introduction

Granulomatosis with polyangiitis (GPA) is a necrotizing small-vessel vasculitis characterized by granulomatous inflammation involving the respiratory tract and kidneys [1,2]. It is commonly associated with proteinase 3-antineutrophil cytoplasmic antibodies (PR3-ANCAs) and can present with a wide spectrum of clinical manifestations, ranging from localized disease to life-threatening systemic involvement. Pulmonary involvement is common and may include nodules, masses, or diffuse alveolar hemorrhage (DAH), which represents a severe and potentially fatal complication [3,4]. Renal disease typically manifests as rapidly progressive glomerulonephritis [5,6].

In some cases, GPA may mimic malignancy radiologically, particularly when presenting with pulmonary masses in patients with risk factors such as smoking [4]. The coexistence of infection further complicates the clinical picture, creating a significant diagnostic dilemma among infection, malignancy, and vasculitis, which may delay appropriate immunosuppressive therapy.

## Case presentation

**Figure 1:**
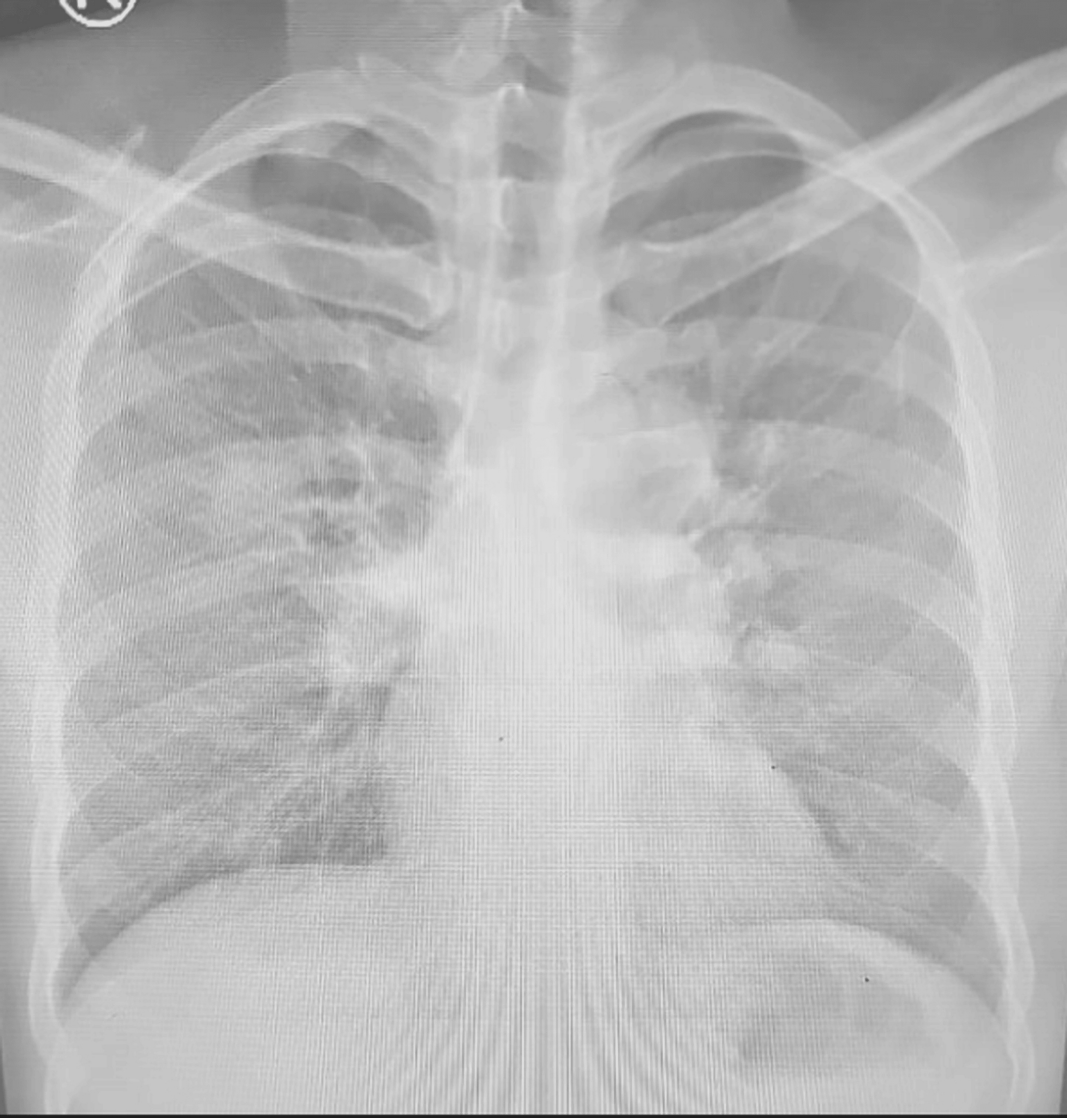
Chest X-ray on admission showing mediastinal widening suspicious for mediastinal masses, with no definite pneumonic consolidation

A 39-year-old male smoker with a significant family history of lung cancer presented with a 20-day history of fever, weight loss, dyspnea, and hemoptysis. Initial investigations suggested an infectious etiology. Sputum cultures grew* Pseudomonas aeruginosa* and *Haemophilus influenzae*. However, the lack of significant clinical improvement despite appropriate antimicrobial therapy raised suspicion for an alternative diagnosis. Chest X-ray on admission demonstrated mediastinal widening suspicious for mediastinal masses, without clear pneumonic consolidation (Figure 1).

**Figure 2:**
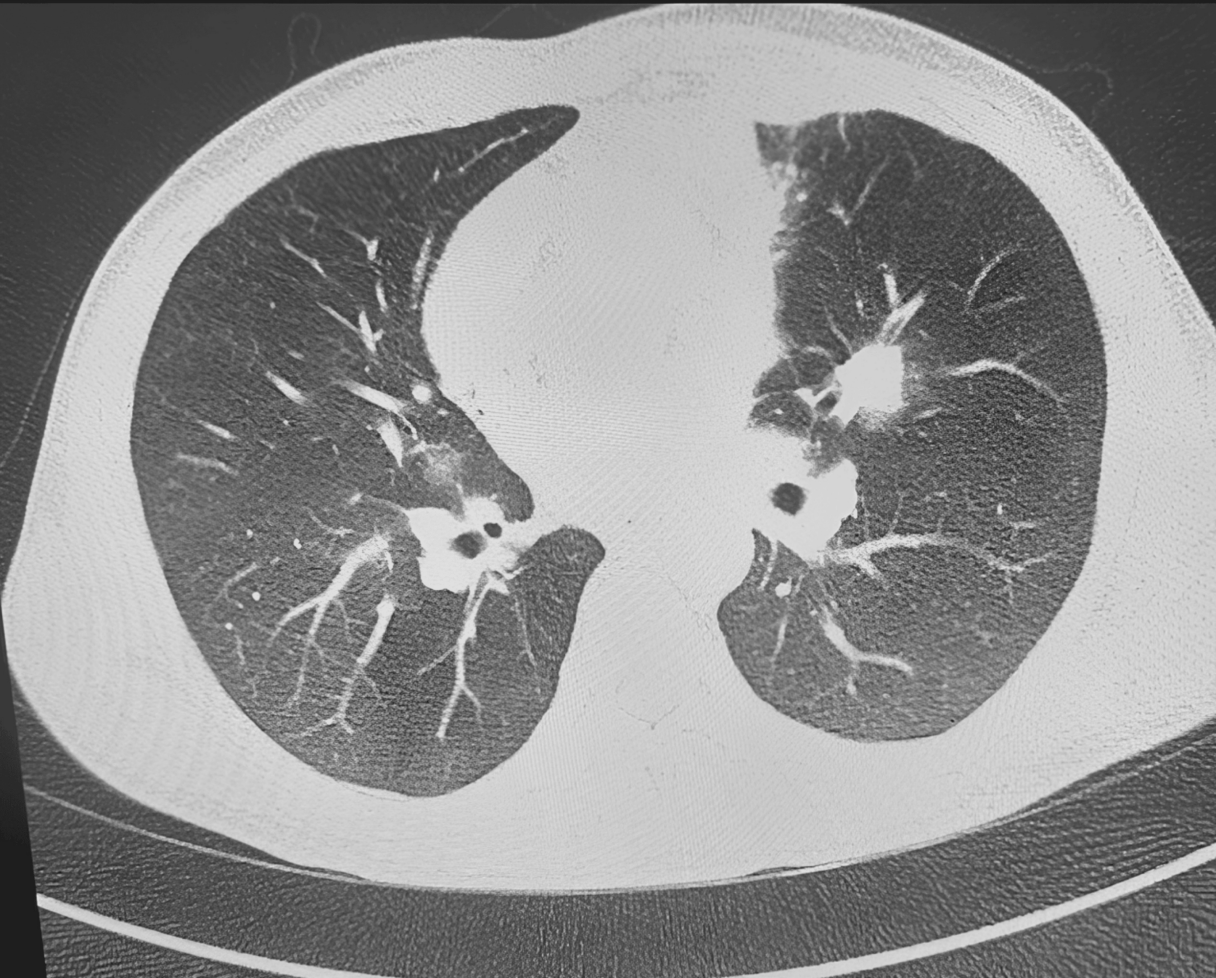
CT pulmonary angiography showing bilateral pulmonary soft tissue masses with no evidence of pulmonary embolism

Due to persistent hemoptysis, CT pulmonary angiography was performed, which excluded pulmonary embolism but revealed bilateral pulmonary soft tissue masses (Figure 2).

**Figure 3:**
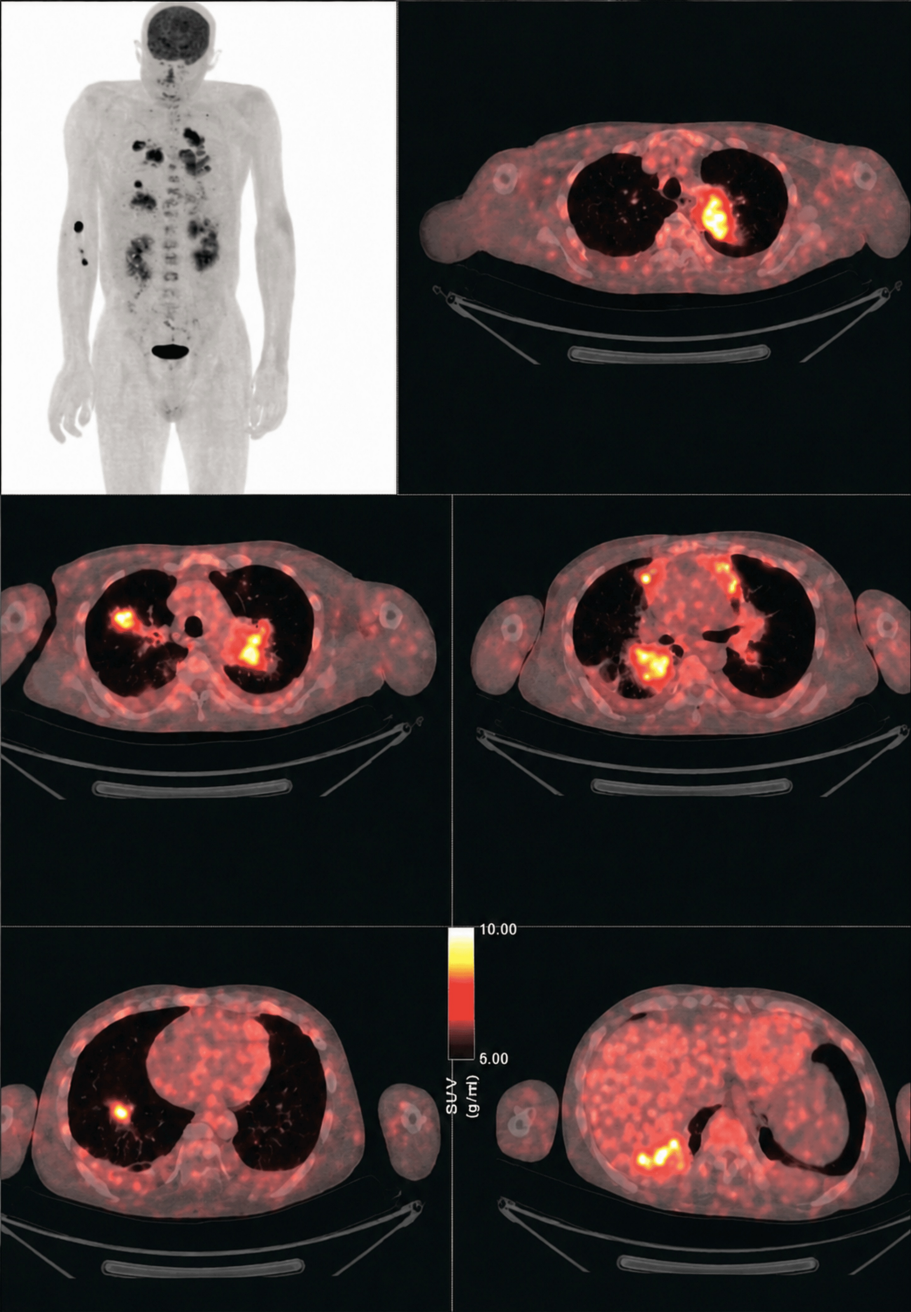
PET-CT demonstrating multiple hypermetabolic lung lesions

Given the patient’s smoking history and family history, malignancy was strongly suspected. PET-CT demonstrated multiple hypermetabolic lung lesions (Figure 3), while CT abdomen was unremarkable.

**Figure 4:**
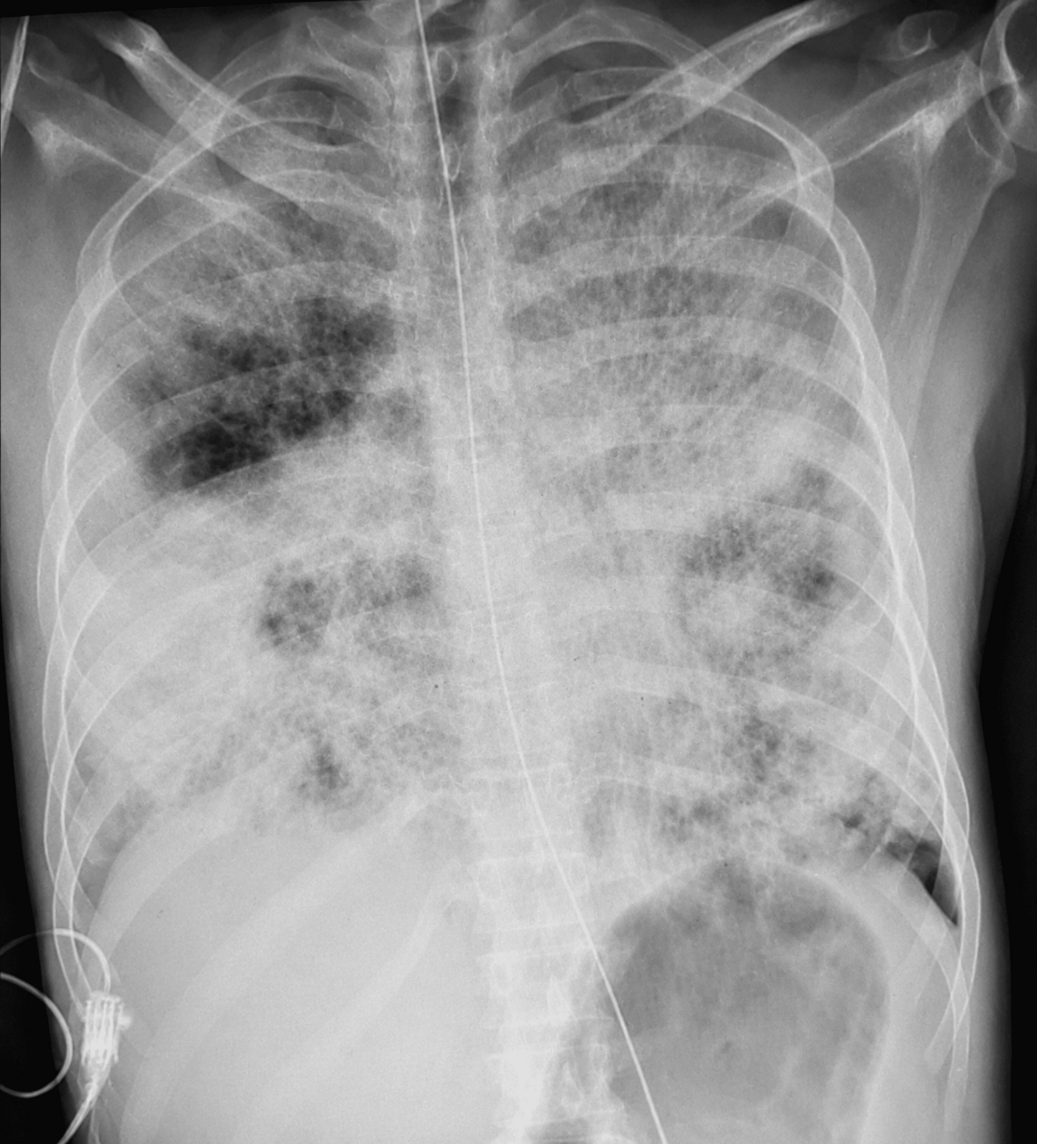
Chest X-ray during deterioration showing bilateral diffuse opacification

Given the radiological findings and clinical context, malignancy remained a strong differential diagnosis at this stage. Further virological screening revealed cytomegalovirus (CMV) viremia (~745 copies/mL), and the patient received antimicrobial therapy, including antiviral coverage with ganciclovir. The patient subsequently deteriorated with massive hemoptysis and a drop in hemoglobin from 11.5 to 7.5 g/dL. He required blood transfusion and ICU admission. Chest X-ray at deterioration showed bilateral diffuse opacification (Figure 4).

**Figure 5:**
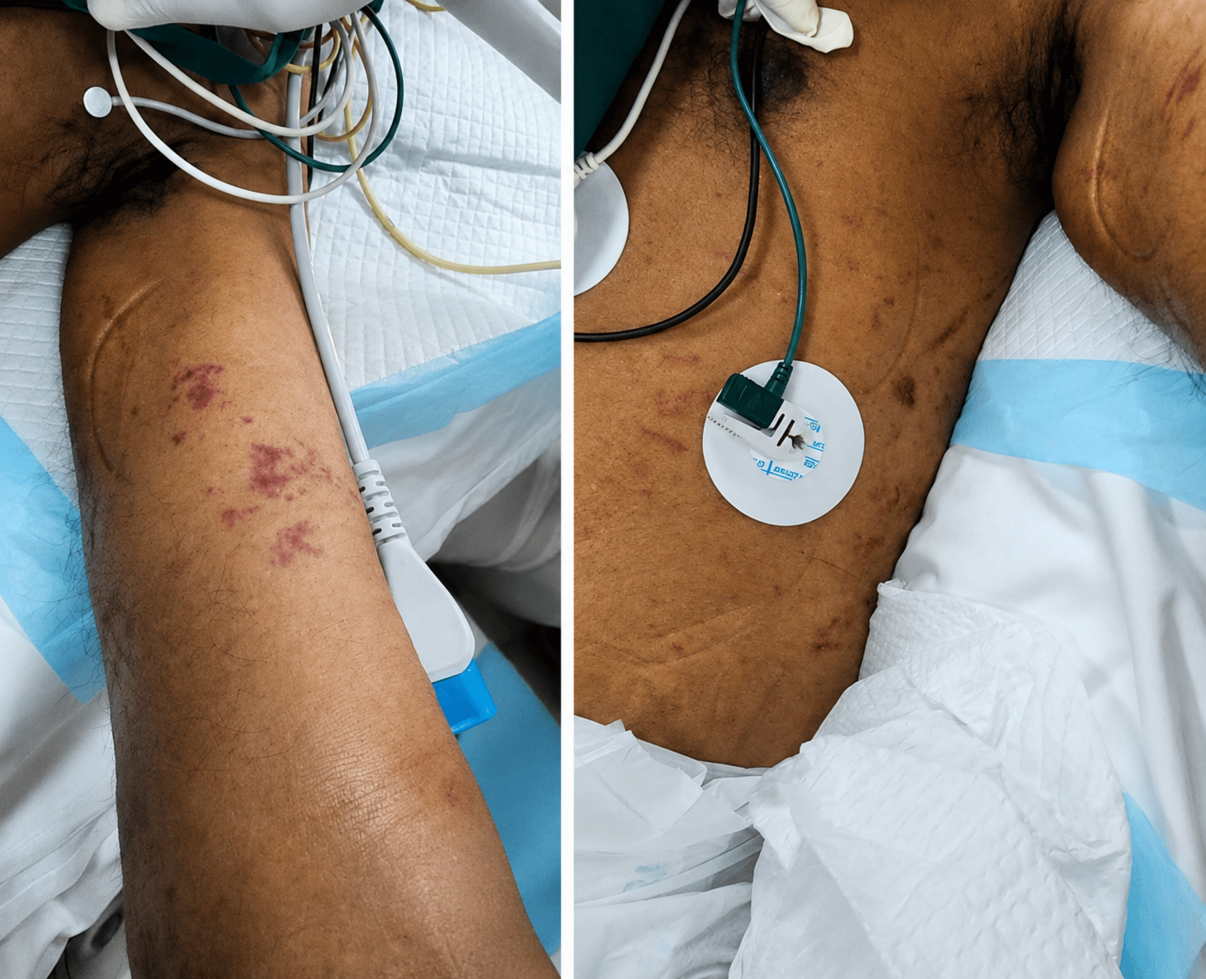
Clinical image of vasculitic skin rash

He required mechanical ventilation and developed acute kidney injury (creatinine ~400 µmol/L), requiring dialysis. Rheumatological evaluation revealed a vasculitic skin rash (Figure 5).

**Figure 6:**
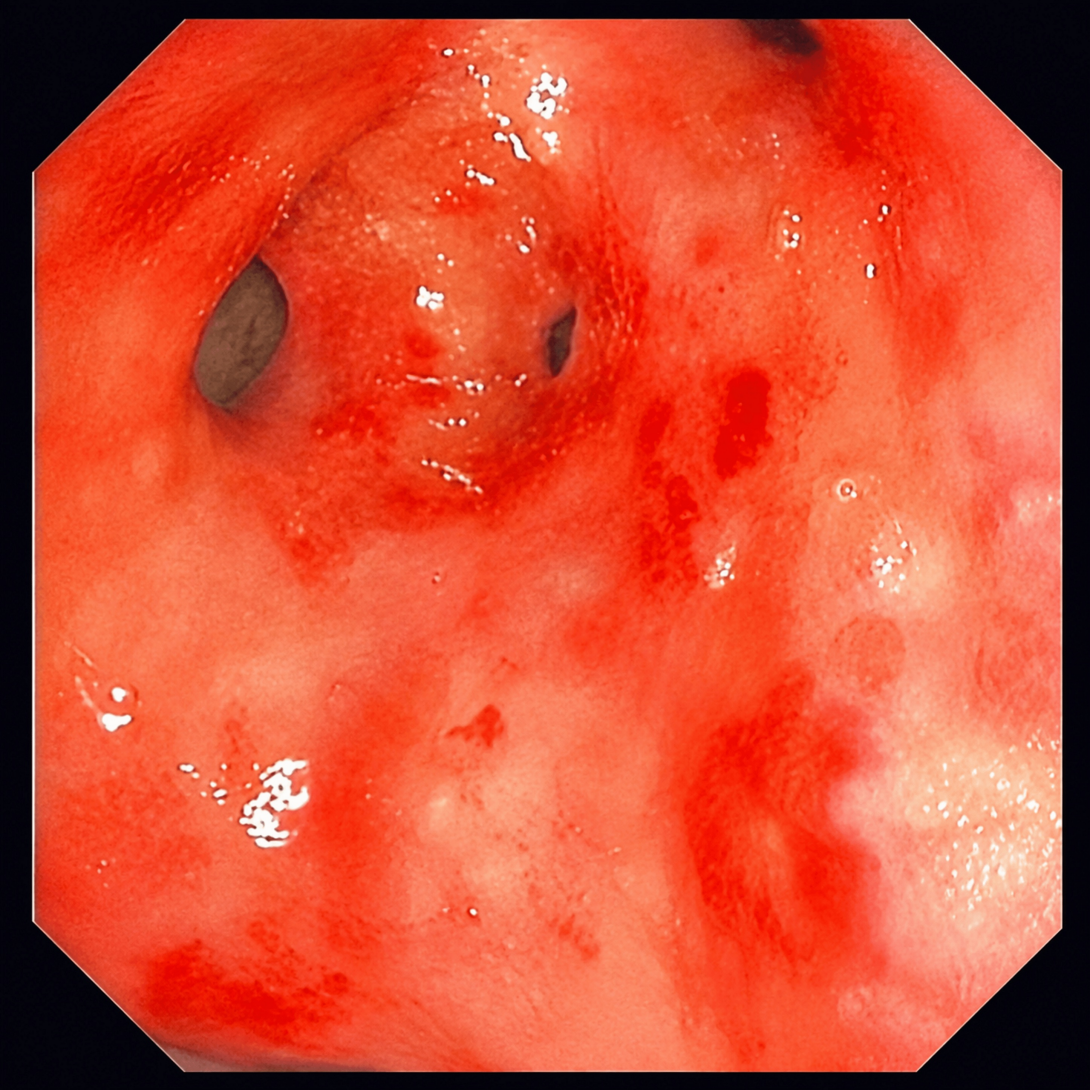
Bronchoscopy showing diffuse hyperemic mucosa

Skin biopsy confirmed leukocytoclastic vasculitis. Laboratory findings demonstrated positive PR3/c-ANCA, low complement C3, and active urinary sediment with red blood cell casts. Bronchoscopy showed diffuse hyperemic mucosa (Figure 6), and bronchoalveolar lavage confirmed DAH with hemosiderin-laden macrophages.

As the clinical picture evolved with combined pulmonary and renal involvement, a pulmonary-renal syndrome was suspected. A diagnosis of GPA presenting as pulmonary-renal syndrome was established.

The patient received pulse methylprednisolone followed by IV pulse cyclophosphamide [7]. Due to severe disease, seven sessions of plasma exchange were performed [3,8].

**Figure 7:**
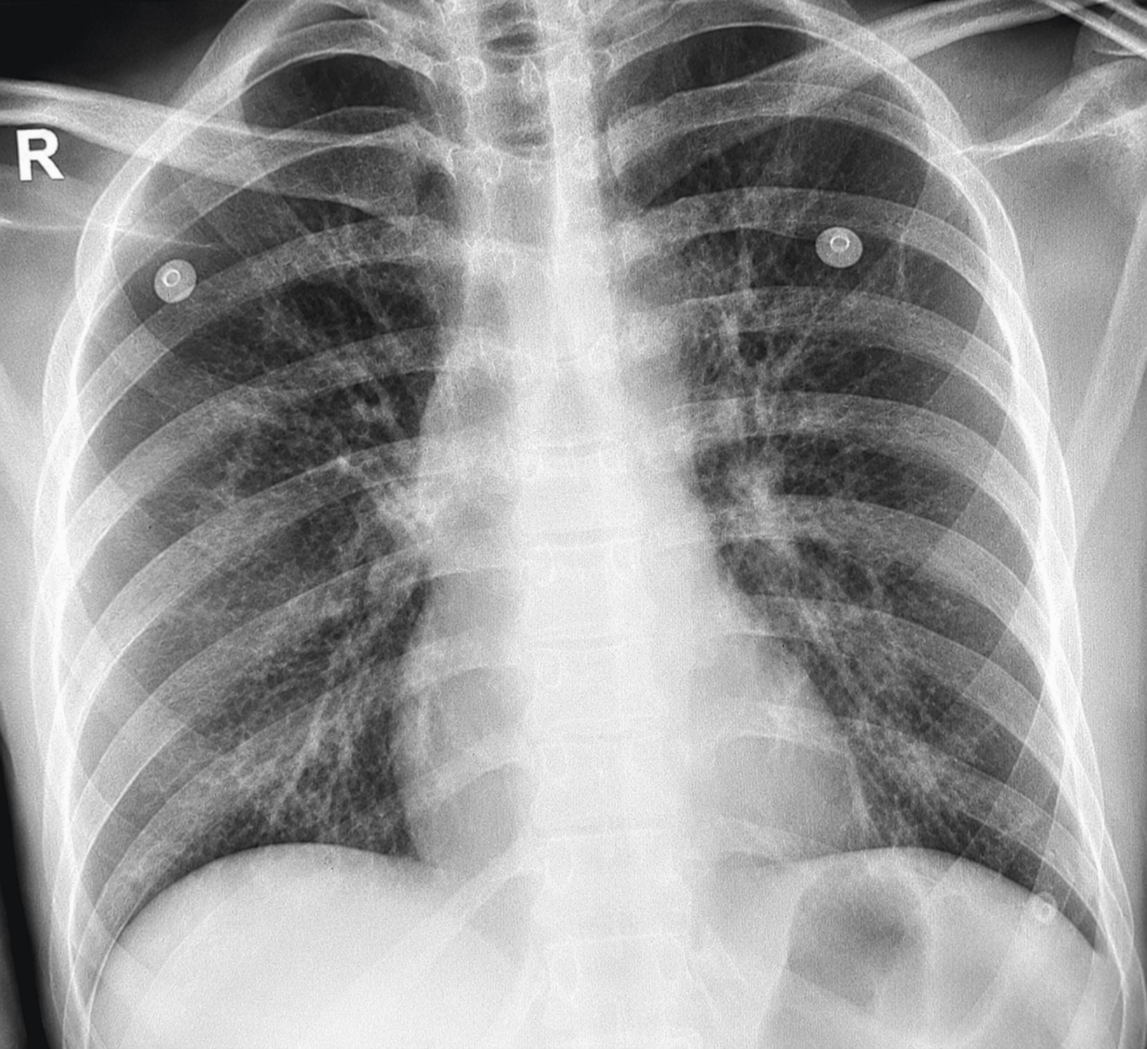
Follow-up chest X-ray showing interval resolution of bilateral opacities with clear lung fields

The patient improved significantly, with resolution of hemoptysis, successful extubation, and normalization of renal function. Follow-up chest X-ray demonstrated interval resolution of bilateral opacities with clear lung fields (Figure 7).

A summary of the clinical course is presented in Table 1.

**Table 1:** Clinical timeline AKI, acute kidney injury; ANCA, antineutrophil cytoplasmic antibody; CMV, cytomegalovirus; GPA, granulomatosis with polyangiitis; PE, pulmonary embolism

Time point	Clinical events
Day 0	Admission with fever, weight loss, hemoptysis
Early admission	Positive sputum cultures, treated as pneumonia
Early imaging	Chest X-ray: mediastinal widening
Day 6	CT pulmonary angiography: no PE, bilateral lung masses
Day 10	PET-CT: multiple hypermetabolic lung lesions
Day 12	CMV viremia detected, ganciclovir started
Day 19	Massive hemoptysis and hemoglobin drop
ICU admission	Mechanical ventilation and transfusion
ICU course	AKI requiring dialysis
Rheumatology	ANCA positive with vasculitic rash
Diagnosis	GPA
Treatment	Steroids, cyclophosphamide, and plasma exchange
Outcome	Recovery and discharge

## Discussion

This case illustrates a complex presentation of GPA with overlapping features of infection and malignancy. The presence of confirmed bacterial infection and CMV viremia initially supported an infectious diagnosis, while radiological findings suggested malignancy, particularly in the context of smoking and family history [4]. This overlap contributed to diagnostic uncertainty, a scenario frequently reported in ANCA-associated vasculitis, where overlapping clinical features may obscure early recognition and delay appropriate treatment [4].

The development of pulmonary-renal syndrome with DAH represented the key diagnostic turning point. DAH is a severe manifestation of ANCA-associated vasculitis associated with significant morbidity [3]. The diagnosis was supported by PR3-ANCA positivity and histopathological findings, consistent with established classification criteria [2].

Management required careful balancing between immunosuppressive therapy and infection control. Current guidelines recommend high-dose glucocorticoids combined with cyclophosphamide or rituximab in patients with organ- or life-threatening disease [5,6].

IV pulse cyclophosphamide provides effective disease control with reduced cumulative toxicity [7]. Plasma exchange remains controversial following the PEXIVAS trial but may still be considered in selected patients with severe renal impairment or pulmonary hemorrhage [3,8].

A key diagnostic challenge in this case was distinguishing among infection, malignancy, and vasculitis. While microbiological findings initially supported an infectious etiology, the lack of sustained clinical improvement and the presence of atypical features raised suspicion for alternative diagnoses. Radiological findings suggested malignancy, particularly in the context of smoking history; however, the subsequent development of pulmonary-renal syndrome pointed toward an underlying vasculitic process. This case highlights the importance of maintaining a broad differential diagnosis and integrating clinical, radiological, and laboratory data in complex presentations. In this case, delayed recognition of ANCA-associated vasculitis in the setting of overlapping infectious and malignant features underscores a significant diagnostic challenge, emphasizing the importance of early diagnosis and prompt initiation of immunosuppressive therapy [9].

## Conclusions

This case highlights the diagnostic challenge of GPA presenting with overlapping infectious and malignant features, which may lead to significant delays in diagnosis. The coexistence of confirmed infection and radiological findings suggestive of malignancy initially obscured the underlying vasculitic process. The subsequent development of pulmonary-renal syndrome with DAH represented a critical turning point that prompted appropriate diagnostic evaluation. This case underscores the importance of maintaining a high index of suspicion for ANCA-associated vasculitis in patients who fail to respond to appropriate antimicrobial therapy, as early recognition and timely initiation of immunosuppressive treatment are essential for improving clinical outcomes.
